# Access to carbon nanofiber composite hydrated cobalt phosphate nanostructure as an efficient catalyst for the hydrogen evolution reaction

**DOI:** 10.3389/fchem.2023.1129133

**Published:** 2023-02-23

**Authors:** Imtiaz Ahmed, Rathindranath Biswas, Rohit Sharma, Vishal Burman, Krishna Kanta Haldar

**Affiliations:** Department of Chemistry, Central University of Punjab, Bathinda, India

**Keywords:** water splitting, composite, carbon nanofibers, cobalt phosphate, HER

## Abstract

Attractive technology for producing sustainable hydrogen with water electrolyzers was foreseen as one of the most promising ways to meet the increasing demands of renewable resources and electricity storage. Mainly used for the efficient generation of H_2_, water electrolysis involving hydrogen evolution reactions (HERs) depends on efficient and affordable electrocatalysts. Hydrogen is an effective fuel that can be produced by splitting water. Hence, the search for highly efficient HER catalysts is a major challenge as efficient hydrogen evolution catalysts are sought to replace catalysts such as platinum. Here, we describe a low-cost and highly effective electrocatalyst for the proper incorporation of the HER electrocatalyst with low overpotential, effective charge transfer kinetics, low Tafel slope, and good durability. By using a simple hydrothermal approach to produce Co_3_(PO_4_)_2_.8H_2_O/CNF, it is possible to attach Co_3_(PO_4_)_2_.8H_2_O to the surface of carbon nanofibers (CNFs), which also exhibit remarkable HER activity at an overpotential of 133 mV and produce a current density of 10 mA/cm^2^ and a 48 mV/decade for the Tafel slope. Large electrochemical surface areas and easy charge transfer from Co_3_(PO_4_)_2_.8H_2_O to the electrode through conductive Co_3_(PO_4_)_2_.8H_2_O/CNF composites are the reasons for the improved performance of Co_3_(PO_4_)_2_.8H_2_O/CNF.

## Graphical Abstract

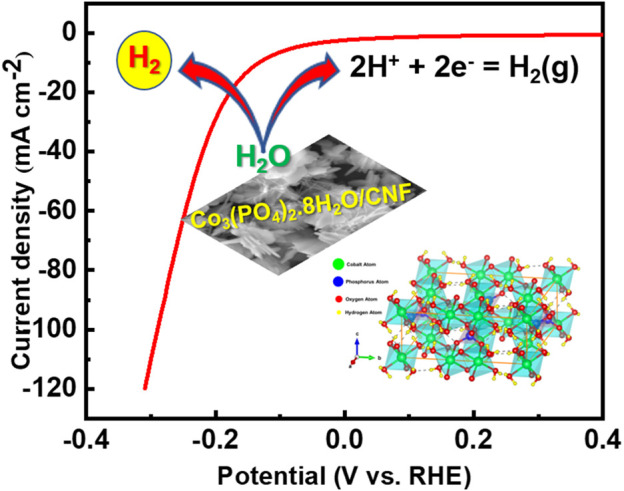

## Introduction

The energy crisis, environmental degradation, and global warming—all driven by the extensive use of fossil fuels—have motivated the production of clean and renewable energy. Currently, due to the accelerating spread of the global energy crisis and the ongoing decay of traditional fossil fuels, a clean, high-energy-density hydrogen economy derived from renewable sources is being extensively studied and developed (Zou and Zhang, 2015). The H_2_ evolution reaction (HER) critically depends on appropriate electrocatalysts such as platinum (Pt) and its alloys, which catalyze the conversion of pairs of protons and electrons to H_2_ at high reaction rates with low overpotentials. However, the high price and relative scarcity of Pt severely limits its widespread use. Therefore, finding reliable and effective alternative catalysts that are geologically abundant is imperative for the future of the hydrogen economy (Gao et al., 2015). The development of an inexpensive HER electrocatalyst, using base elements with superior activity and high stability as a replacement for expensive platinum, has been one of the most pressing goals in recent years. In recent decades, several studies have been conducted to replace noble-metal-based electrocatalysts (Bui et al., 2020).

Because of its ability to catalyze the splitting of water, cobalt (Co) has become an intriguing base metal. The preparation of Co-based composites and complexes using homogeneous molecular catalysts has received much research attention (Anjum et al., 2018; Ahmed et al., 2021; Hu et al., 2021a; Singh et al., 2022a; Singh et al., 2022b). In addition, cobalt-based electrocatalysts (CoP, Co_3_O_4_, CoOOH, CoSe_2_, etc.) have attracted significant attention for a variety of applications, including sensors (Hu et al., 2021b; Sari et al., 2022), supercapacitors (Rovetta et al., 2017; Li et al., 2018), lithium-ion batteries (Khan et al., 2016), and OER (Sun et al., 2021; Biswas et al., 2022; Kubba et al., 2022). Because of their strong electrochemical activity, cobalt phosphates have attracted much attention in recent decades and have been used extensively in electrochemical energy storage and as electrocatalysts for water splitting (Shu et al., 2018; Majhi and Yadav, 2021a). An inductive influence due to the presence of (PO_4_)_3_ groups means that the redox coupling of the transition metal is significantly higher than that of the comparable oxide. In addition, the excellent ionic conductivity of the large (PO_4_)_3_ units creates open pathways that can facilitate rapid ionic migration (Samal et al., 2016). Carbon-based materials (carbon nanofibers and carbon nanotubes) have attracted significant interest in an attempt to improve the long-term stability of the catalysts, while transition metal oxides (Zhao et al., 2018; Zhu et al., 2019; Ahmed et al., 2021; Majhi and Yadav, 2021b), sulfides (Biswas et al., 2021; Majhi and Yadav, 2021c; Sun et al., 2021), and phosphates (Ahmed et al., 2022; Majhi and Yadav, 2022a; Majhi and Yadav, 2022b) of other transition metals have been used as binding and support materials due to their excellent corrosion resistance, good conductivity, and adjustable chemical surface properties. Carbon-based materials have received significant attention due to their adaptable surface chemistry, strong conductivity, and excellent corrosion resistance. Therefore, to achieve highly efficient overall water splitting to produce clean H_2_, a rational design of electrocatalysts is required. This must account for processing costs, catalytic activity, and long-term stability (Woo et al., 2020).

In this work, we synthesized a hydrated phosphate-based carbon-nanofiber-supported material (Co_3_(PO_4_)_2_.8H_2_O/CNFs) to study the HER performance of the catalyst in acidic media. Hydrated cobalt phosphate with carbon nanofibers has shown excellent stability over 24 h, implying its superior stability during the HER reaction. The composite helps increase the highly electroactive surface area, high conductivity, and vertical growth relative conducting CNFs, exposing a high density of edge phosphate. Other factors that increase the activity of Co_3_(PO_4_)_2_.8H_2_O/CNFs are high current density, low Tafel slope, and low charge transfer resistance, which are 133 mV, 48 dec/cm^1^, and 43.04, respectively. The developed catalyst showed electrocatalytic performance comparable to commercial Pt/C in the acidic HER medium.

## Chemicals used

### Materials

Cobalt (II) chloride (CoCl_2_), diammonium hydrogen phosphate (NH_4_)_2_HPO_4_, commercially available CNF, ethanol (99.9% AR grade), and potassium hydroxide (KOH) were purchased from Loba Chemie, and platinum carbon (Pt/C) and 5% Nafion™ 117 solution were purchased from Sigma-Aldrich. All chemicals were stored in a dry place and used without any further purification.

## Experimental section

### Preparation of Co_3_(PO_4_)_2_.8H_2_O/CNF

Co_3_(PO_4_)_2_.8H_2_O/CNFs were prepared using a facile hydrothermal method. In a typical synthesis, 259.6 mg of CoCl_2_ was dispersed in DIH_2_O and then 45 mg of CNF (commercial) was added, followed by stirring for 10 min. The reaction mixture was transferred to a Teflon-lined autoclave for hydrothermal treatment at 180°C for 24 h. The sample was collected by centrifugation and washed several times with both distilled water and ethanol to remove undesired species, and the product was dried in an oven at 70°C for 12 h.

## Result and discussion

### X-ray diffraction

**FIGURE 1:**
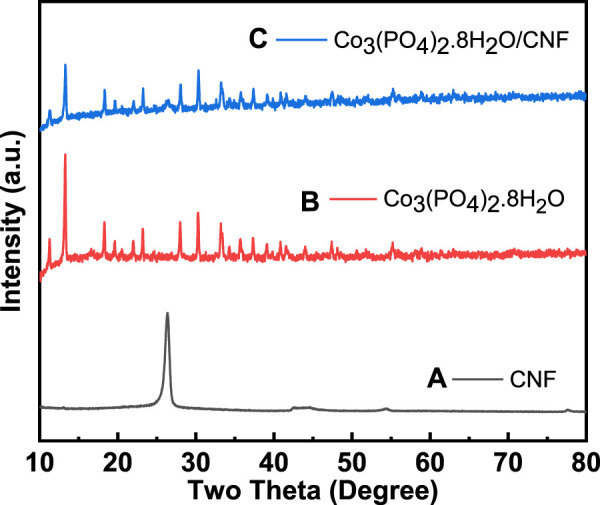
**(A)** XRD pattern of CNF, **(B)** Co_3_(PO_4_)_2_.8H_2_O, and **(C)** Co_3_(PO_4_)_2_.8H_2_O/CNF composite structure.

**FIGURE 2:**
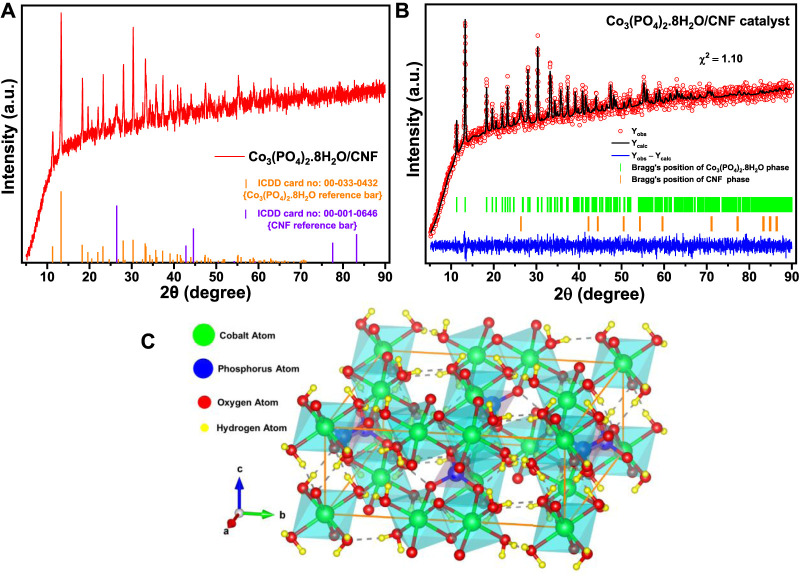
**(A)** XRD pattern of the Co_3_(PO_4_)_2_.8H_2_O/CNF catalyst, **(B)** Rietveld-refined XRD pattern of the Co_3_(PO_4_)_2_.8H_2_O/CNF catalyst, and **(C)** VESTA-generated polyhedral ball–stick model crystal structure of the monoclinic crystal lattice of the Co_3_(PO_4_)_2_.8H_2_O phase with unit cell volume 603.095581 Å^3^ (drawn with solid orange lines), where a = 10.02100 Å, b = 13.32390 Å, c = 4.67245 Å, *α* = *γ* = 90°, and *β* = 104.8240°.

To identify the crystallographic phases and the formation of synthesized Co_3_(PO_4_)_2_.8H_2_O/CNF, along with Co_3_(PO_4_)_2_.8H_2_O and CNF, an X-ray diffraction (XRD) analysis was conducted; the results are shown in Figure 1 and Figure 2. The XRD pattern of the hydrothermal synthesized (Co_3_(PO_4_)_2_.8H_2_O/CNF) catalyst is intact and well-matched with the ICSD card no: 00-033-0432 of hydrated cobalt phosphate, and most prominent broad diffraction peaks assigned to the (002) plane of CNF at the 2θ position of 26.35° are present (Figure 1A). This outcome primarily suggests the presence of hydrate molecules in the hydrothermally synthesized Co_3_(PO_4_)_2_.8H_2_O/CNF catalyst. The XRD pattern exhibited dominant peaks at 2θ with values of 11.26°, 13.26°, 18.28°, 19.62°, 21.97°, 23.19°, 27.97°, 30.31°, 33.17°, 33.36°, 35.67°, 37.31°, 40.79°, and 41.53°, corresponding to the lattice planes of (110), (0 2 0), (2 0 0), (−1 0 1), (1 3 0), (1 0 1), (0 3 1), (2 1 1), (−3 2 1), (−1 4 1), (1 4 1), (3 0 1), (−3 4 1), and (−2 5 1), respectively. The diffraction patterns of Co_3_(PO_4_)_2_.8H_2_O (Figure 1B) indexed well with the monoclinic phase 12/m space group standard patterns, and no other peaks were detected in the XRD patterns. However, the XRD pattern of Co_3_(PO_4_)_2_.8H_2_O/CNF (Figure 1C) showed peaks corresponding to both Co_3_(PO_4_)_2_.8H_2_O and CNF, indicating the formation of a Co_3_(PO_4_)_2_.8H_2_O/CNF hybrid structure. The observed peaks had high intensities, clearly showing that the as-prepared samples were highly crystalline. No other impurities were found. The levels of the high-intensity peaks are highlighted in the composite spectra.

Furthermore, for comprehensive conclusiveness, we performed the Rietveld refinement of the PXRD pattern of Co_3_(PO_4_)_2_.8H_2_O/CNF (Figure 2) by FullProf Suite software. The crystal structure was generated using a refined CIF file in VESTA software. As shown by the atomic positions given in Supplementary Table S2, we clearly identified the presence of hydrogen atoms along with the copper, phosphorus, and oxygen atoms. These hydrogen atoms came from the H_2_O molecules conjugated with the Co_3_(PO_4_)_2_ lattice. Now, the Co_3_(PO_4_)_2_.8H_2_O lattice crystallizes into a 2D monoclinic structure with the space group C12/m 1. It is composed of two Co_3_P_2_(OH)_16_ sheets with preferential orientation in the (0 1 0) direction. Among the two inequivalent Co^2^⁺ sites, the first site (corresponding to the Co1 atom at the 2a Wyckoff site) is bonded to six O^2^⁻ atoms in the CoO₆ octahedron, which shares corners with the two equivalent PO₄ tetrahedra. There are two shorter (2.0624 Å) and four longer (2.1646 Å) Co–O bond lengths. At the second Co^2^⁺ site (corresponding to the Co_2_ atom at the 4g Wyckoff site), Co^2^⁺ is bonded to six O^2^⁻ atoms to form a CoO₆ octahedron that shares corners with four equivalent PO₄ tetrahedra and an edge with one CoO₆ octahedron. The Co–O bond distances range between 2.0755 and 2.1822 Å. P⁵⁺ is bonded to four O^2^⁻ atoms to occupy the PO₄ tetrahedral site that shares corners with five CoO₆ octahedra. The P–O bond distances range from 1.5529 to 1.5910 Å. There are five inequivalent O^2^⁻ sites; among them, at the first O^2^⁻ site (corresponding to the O1 atom), O^2^⁻ is bonded in a nine-coordinate geometry to one Co^2^⁺ and eight H^1^⁺ atoms (3 H1, 3 H2, H3, and H4). At the second O^2^⁻ site (corresponding to the O2 atom), O^2^⁻ is bonded in a six-coordinate geometry to one Co^2^⁺ atom and five H^1^⁺ atoms (2 H1, 2 H3, and H4). At the third O^2^⁻ site (corresponding to the O3 atom), O^2^⁻ is bonded in a four-coordinate geometry to one Co^2^⁺, one P⁵⁺, and two equivalent H^1^⁺ atoms (H3 and H4). At the fourth O^2^⁻ site (corresponding to the O4 atom), O^2^⁻ is bonded in a three-coordinate geometry to two equivalent Co^2^⁺ atoms and one P⁵⁺ atom. At the fifth O^2^⁻ site (corresponding to the O5 atom), O^2^⁻ is bonded in a six-coordinate geometry to one Co^2^⁺, one P⁵⁺, and four equivalent H^1^⁺ atoms (H2). Among the four inequivalent H^1^⁺ sites, at the first H^1^⁺ site (corresponding to the H1 atom), H^1^⁺ is bonded in a five-coordinate single-bond geometry to five O^2^⁻ atoms (3 O1 and 2 O2), with a shorter H–O bond length of 0.8835 Å (O2) and a longer H–O bond length of 2.8099 Å (O1). At the second H^1^⁺ site (corresponding to the H2 atom), H^1^⁺ is bonded in a five-coordinate single-bond geometry to five O^2^⁻ atoms (3 O1 and 2 O5), with a shorter H–O bond length of 0.9470 Å (O1) and a longer H–O bond length of 2.6647 Å (O5). At the third H^1^⁺ site (corresponding to the H3 atom), H^1^⁺ is bonded in a four-coordinate single-bond geometry to four O^2^⁻ atoms (O1, 2 O2, and 2 O3), with a shorter H–O bond length of 0.8675 Å (O2) and a longer H–O bond length of 2.8224 Å (O1). At the fourth H^1^⁺ site (corresponding to the H4 atom), H^1^⁺ is bonded in a three-coordinate single-bond geometry to three inequivalent O^2^⁻ atoms (O1, O2, and O3) with H–O bond lengths of 0.6671 Å (O1), 2.7845 Å (O1), and 2.1287 Å (O5). Thus, we can conclude from the crystal structure symmetry that the Co_3_(PO_4_)_2_ phase contains water molecules in the Co_3_(PO_4_)_2_.8H_2_O/CNF composite system as we synthesized it in hydrothermal treatment and because at low temperature, the Co_3_(PO_4_)_2_ phase is stable in its ortho-hydrate due to its thermodynamical preferences.

**FIGURE 3:**
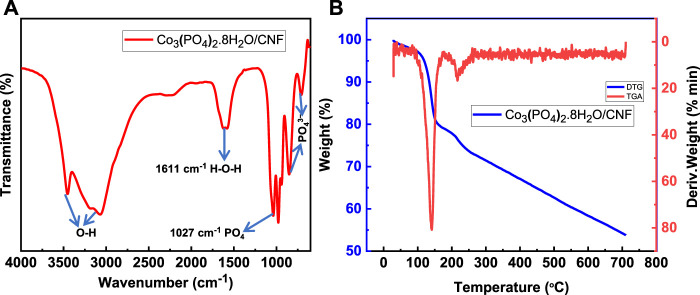
**(A)** FTIR analysis, and **(B)** TGA (blue, left axis) and DTG (red, right axis) analysis of Co_3_(PO_4_)_2_·8H_2_O.

Fourier-transform infrared spectroscopy (FTIR), thermogravimetric analysis (TGA), and derivative thermogravimetric (DTG) analysis are presented in Figure 3. The FTIR spectrum (Figure 3A) demonstrates that the stretching of the P–O–P linkages is attributed to the peaks at 714 cm^−1^ and 855 cm^−1^ and shows the vibrational mode of PO_4_^3-^. The strong absorption asymmetric vibration mode of the PO_4_ group was observed at 1027 cm^−1^. The characteristic band at 1611 cm^−1^ represents the bending vibration mode of a water molecule (H–O–H). Adsorbed water is represented by a broad absorption band from 3051 to 3448 cm^−1^ and can be assigned to the vibrational bond of O–H. These results indicate that the deposited material contains structural water and formation of hydrous cobalt phosphate (Co_3_(PO_4_)_2_.8H_2_O)/CNF.

At higher temperatures and treatment in an inert atmosphere, the cobalt cation and the phosphate anion reinstall a crystal structure. The removal of coordinated water molecules is also confirmed by thermal derivative thermogravimetric (DTG) and gravimetric analysis (TGA), as shown in Figure 3B. The thermogram of the Co_3_(PO_4_)_2_.8H_2_O/CNF material exhibits two distinct loss stages. The first step shows the initial 4.8% weight loss in the temperature range of 105 °C; in the second stage, a 19.3% weight loss is observed in the temperature range of 106°C–153 C due to the loss of water. The total weight loss of the sample was found to be 46.2% when the temperature was up to 700°C.

### Field emission scanning electron microscope (FE-SEM)

**FIGURE 4:**
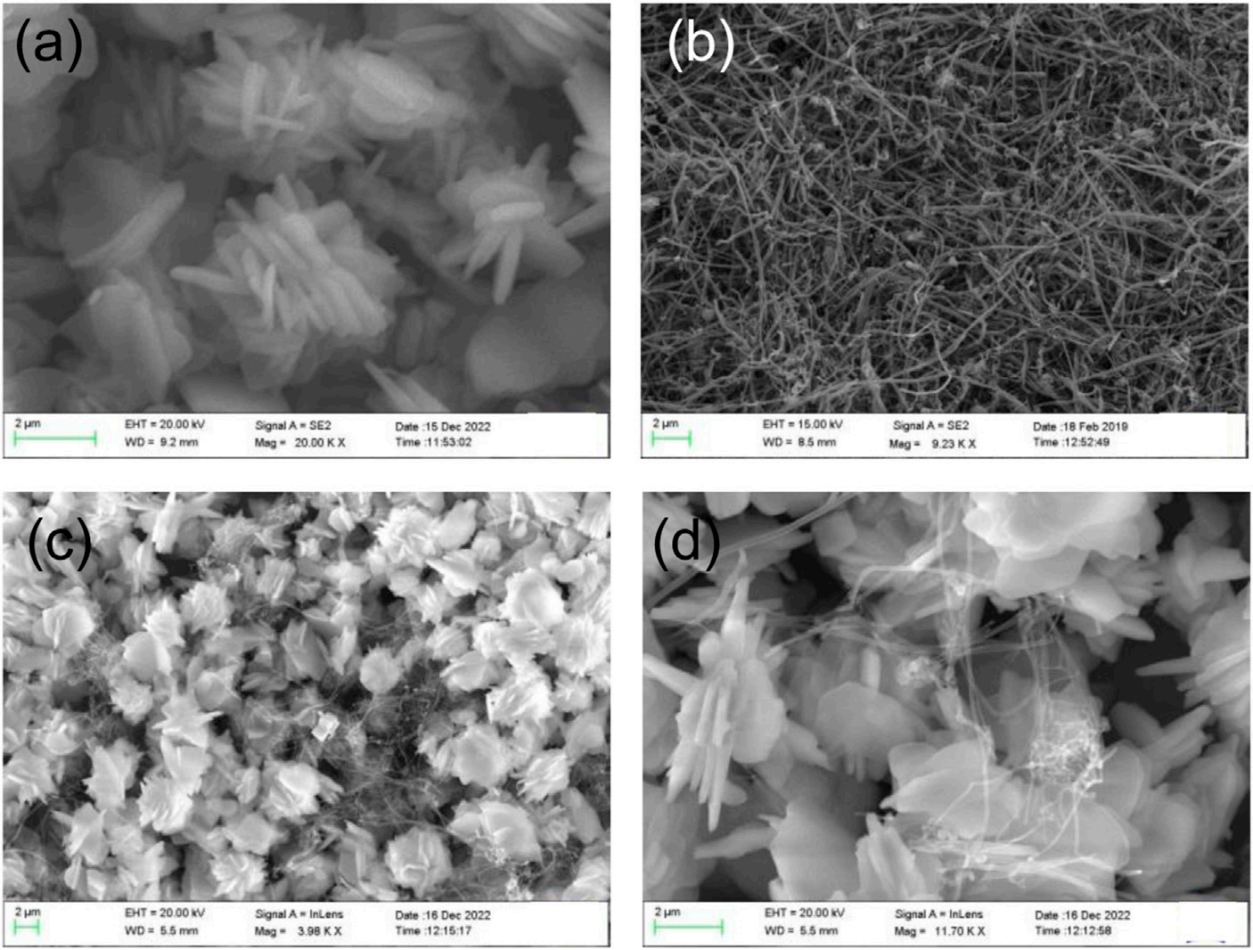
**(A)** SEM images of Co_3_(PO_4_)_2_.8H_2_O, **(B)** CNF, and **(C,D)** Co_3_(PO_4_)_2_.8H_2_O/CNF composite flower.

**FIGURE 5:**
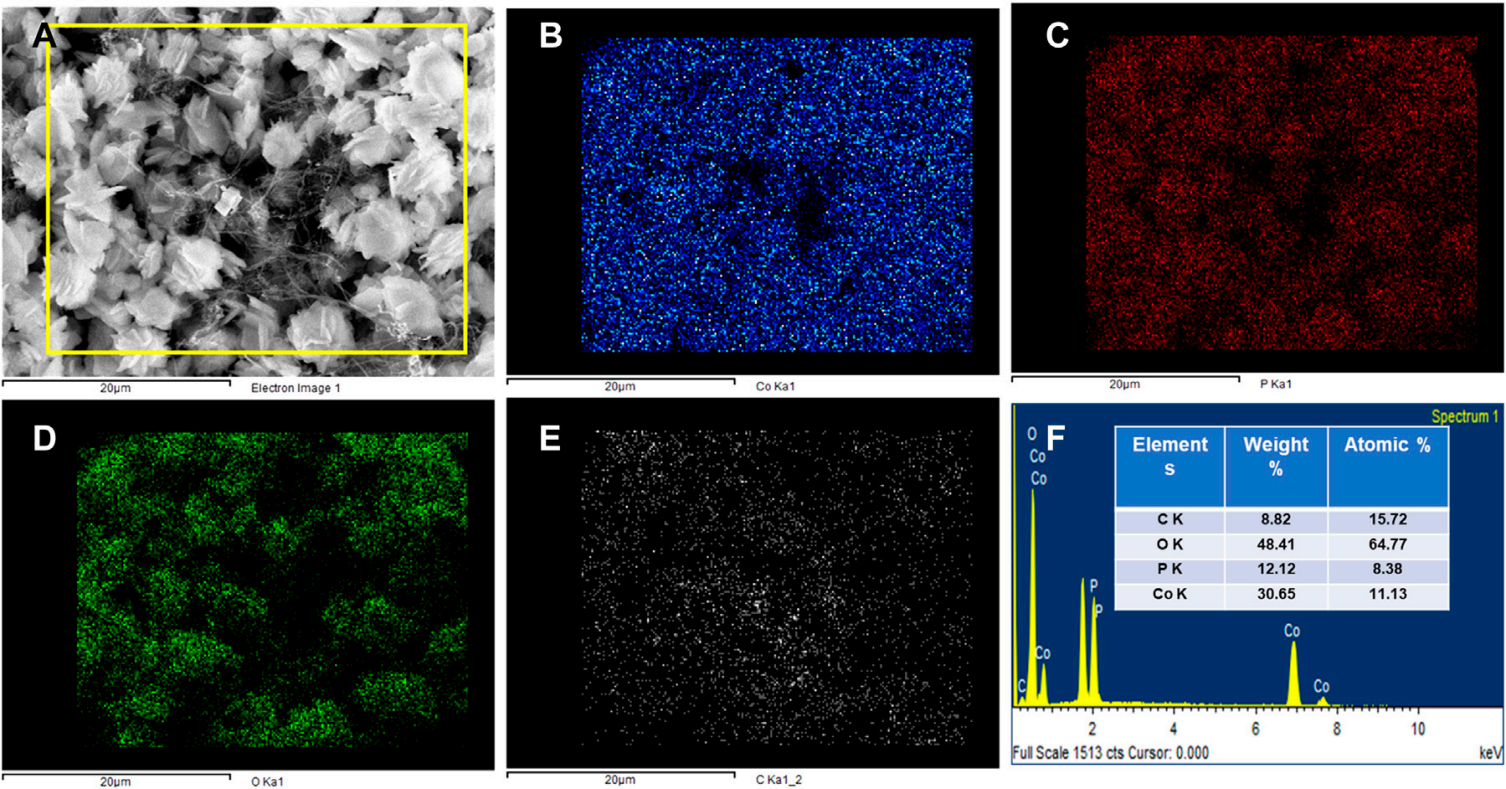
**(A)** Mapping selected image. **(B)** Mapping of Co, **(C)** P, **(D)** O, **(E)** C, and **(F)** EDX of the Co_3_(PO_4_)_2_.8H_2_O/CNF composite flower.

The surface morphology of the Co_3_(PO_4_)_2_.8H_2_O/CNF material was observed by FE-SEM. Typical FE-SEM images of a synthesized sample are shown in Figure 4. The FE-SEM image of Co_3_(PO_4_)_2_.8H_2_O shows a rod-shaped flower (Figure 4A) and resembles CNF with a thread-like morphology (Figure 4B). The Co_3_(PO_4_)_2_.8H_2_O/CNF composite shows that cobalt phosphate flowers are stacked with a uniform and regular structure on the surface of carbon nanofibers; images of the composite are shown in Figures 4C, D. It can be observed that the surface morphology of cobalt phosphate is a tightly packed flower, which may suggest support for effective electron transport. In addition, the elemental color map of the Co_3_(PO_4_)_2_.8H_2_O/CNF composite is shown in Figures 5A–E, where it can be observed that the distribution of Co, O, P, and C is suboptimal, resulting in the exposed substrate observed in these pictures. Energy-dispersive X-ray spectroscopy (EDX) was performed to confirm the elemental composition and is shown in Figure 5F, where all elements are presented while their ratios in weight percentage and atomic percentage are included in Figure 5F. The FE-SEM image of Co_3_(PO_4_)_2_.8H_2_O distribution over CNF, where a large percentage of cobalt phosphate is distributed over CNF, could indicate the increase in conductivity essential for enhancing the electrocatalytic water-splitting reaction.

### X-ray photoelectron spectroscopy (XPS)

**FIGURE 6:**
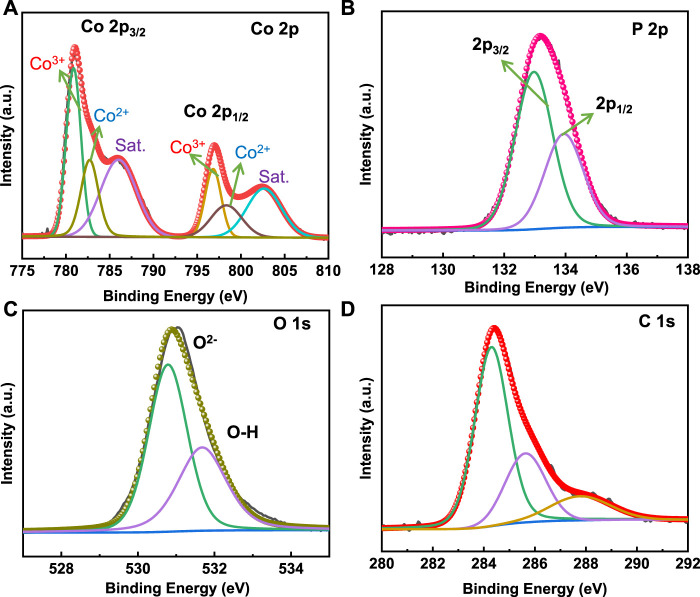
High-resolution deconvoluted XPS spectra of **(A)** Co 2p, **(B)** P 2p, **(C)** O 1s, and **(D)** C 1s energy levels in the Co_3_(PO_4_)_2_.8H_2_O/CNF composite structure.

XPS measurements were performed to validate the chemical composition and oxidation state of Co_3_(PO_4_)_2_.8H_2_O/CNF. The XPS survey spectra of the composite are presented in Supplementary Figure S4, and the high-resolution XPS spectra of Co 2p, P 2p, O 1s, and C 1s are shown in Figure 6. The high-resolution Co 2p orbital consists of two spin-orbit components of 2p_3/2_ and 2p_1/2_ for the Co^2+^ and Co^3+^ states. The two peaks at the binding energies 780.87 and 796.82 eV are assigned to the Co^3+^ state, and 782.71 and 798.34 eV are assigned to the Co^2+^ state for the Co 2p_3/2_ and 2p_1/2_ nuclear levels, respectively. Co 2p shows two satellite peaks of Co 2p_3/2_ and Co 2p_1/2_ core levels (Figure 6A) (Song et al., 2020). The P 2p region of Co_3_(PO_4_)_2_.8H_2_O/CNF shows two characteristic peaks at a binding energy of 132.97 eV and 133.95 eV, corresponding to the 2p_3/2_ nuclear levels and 2p_1/2_, which can be assigned to the phosphate group (Figure 4B). O1s signals are centered at a binding energy of 530.78, and 531.68 eV corresponds to the phosphate oxygen and OH group of H_2_O molecules present in the lattice (Figure 6C). In the C1s spectrum (Figure 6D), the peaks centered at about 284.30, 285.63, and 287.77 eV are indicated on sp^2^-hybridized C-C, C-N, and C-O, respectively (Yuan et al., 2016).

## Electrochemical activity

### HER performance

**FIGURE 7:**
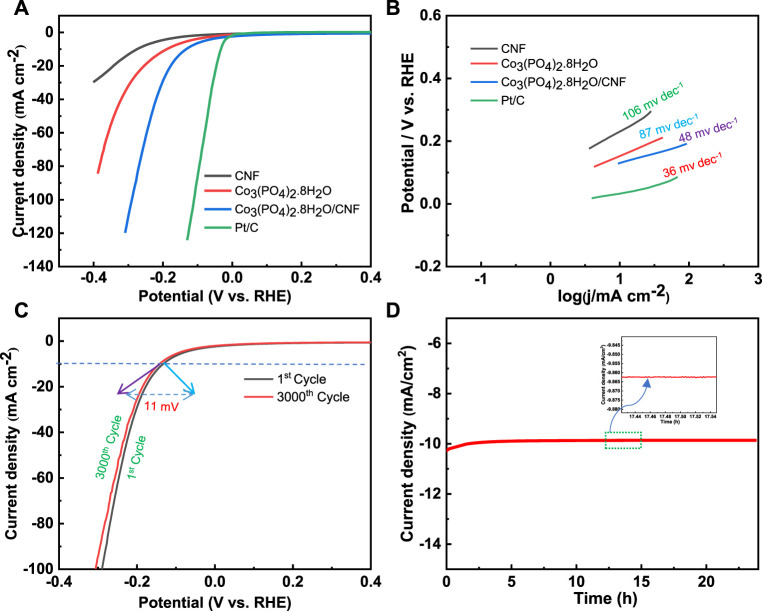
**(A)** Polarization curve (LSV) plot and **(B)** corresponding Tafel plot of Co_3_(PO_4_)_2_.8H_2_O/CNF, Co_3_(PO_4_)_2_.8H_2_O, and CNF, and Pt/C. **(C)** Chronoamperometry method of Co_3_(PO_4_)_2_.8H_2_O/CNF at an overpotential of 133 mV for 24 h. **(D)** Polarization curves of Co_3_(PO_4_)_2_.8H_2_O/CNF of 1st and 3000th cycles of continuous operation.

The linear sweep voltammetry (LSV) curves were measured in the potential window range from 0 V to -1 V *versus* Ag/AgCl for the HER process, with a sampling rate of 10 mV s^−1^ and 0.5 M H_2_SO_4_ solution as the electrolyte. The long-term HER stability test of the catalyst was performed using an Ag/AgCl electrode in the acidic medium. Electrochemical impedance spectroscopy (EIS) was performed in 0.5 M H_2_SO_4_ solution over the frequency range of 100 kHz to 0.1 Hz, at an overpotential of 400 mV. The HER electrocatalytic activity of Co_3_(PO_4_)_2_.8H_2_O/CNF, Co_3_(PO_4_)_2_.8H_2_O, and carbon nanofiber (CNF) was assessed by linear sweep voltammetry (LSV) and compared to data obtained for commercial Pt/C as a reference (Figure 7). Co_3_(PO_4_)_2_.8H_2_O/CNF had the lowest overpotential among all the catalysts, indicating its superior HER activity. Overpotentials of the catalysts are given in Table 1, with the composites Co_3_(PO_4_)_2_.8H_2_O/CNF, Co_3_(PO_4_)_2_.8H_2_O, CNF, and Pt/C having overpotential values of 133 mV, 188 mV, 275 mV, and 32 mV, respectively. Record a current density of 10 mA/cm^2^ (Figure 7A). Using the Volmer and Heyrovsky equation (η = a + b log j, where η is over potential, j is current density, b is Tafel slope and a is constant), we calculated the Tafel slopes of the linear domains to determine the kinetics of the catalysts. We found the Tafel slope values for composites Co_3_(PO_4_)_2_.8H_2_O/CNF, Co_3_(PO_4_)_2_.8H_2_O, CNF, and Pt/C to be 48 mV/dec^1^, 87 mV/dec^1^, 106 mV/dec^1^, and 36 mV/dec^1^ where, the composite Co_3_(PO_4_)_2_.8H_2_O/CNF showed the fastest HER kinetics among all the catalysts prepared (Figure 7B). The long-term cyclic stability of the Co_3_(PO_4_)_2_.8H_2_O/CNF electrocatalyst toward HER activity was also tested, using 0.5 M H_2_SO_4_ solution as the electrolyte for 3000 continuous LSV cycles (Figure 7C). After the stability test, it was found that the overpotential rise was only 8 mV at a current density of 10 mA/cm^2^, proving the superior long-term stability of the Co_3_(PO_4_)_2_.8H_2_O/CNF catalyst to HER activity in the acidic medium. Furthermore, long-term stability was tested by chronoamperometry using 0.5 M H_2_SO_4_ solution as the electrolyte at a constant overvoltage of 133 mV for 24 h (Figure 7D), and the result shows a very high durability with negligible loss in current density of the catalyst.

**TABLE 1:** Summary of the electrochemical HER activity of Co_3_(PO_4_)_2_.8H_2_O/CNF, Co_3_(PO_4_)_2_.8H_2_O, CNF, and Pt/C catalysts in the acidic medium.

Catalyst	Overpotential (mV) at 10 mA cm^−2^	Tafel slope (mV dec^−1^)	R_ct_ (*Ω*) (HER)	R_s_ (*Ω*) (HER)	C_dl_ (mF cm^−2^) (HER)	ECSA (cm^2^) (HER)
Co_3_(PO_4_)_2_.8H_2_O/CNF	133	48	43.04	0.034	3.07	76.97
Co_3_(PO_4_)_2_.8H_2_O	188	87	84.12	0.017	1.66	41.7
CNF	275	106	136.19	0.108	0.83	20.96
Pt/C	32	36	-	-	-	-

**FIGURE 8:**
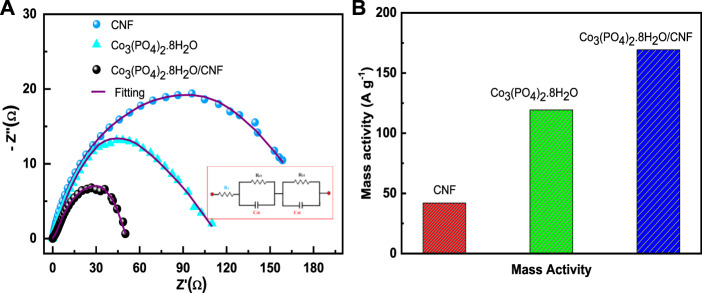
**(A)** Electrochemical impedance spectroscopy (EIS) plots and **(B)** mass activity of the catalysts.

Figure 8A represents electrochemical impedance spectroscopy (EIS), where Co_3_(PO_4_)_2_.8H_2_O/CNF is characterized by the smallest Nyquist plot radius, followed by Co_3_(PO_4_)_2_.8H_2_O and CNF, indicating a lower charge transfer resistance for indicating these three electrocatalysts. The charge transfer resistances of Co_3_(PO_4_)_2_.8H_2_O/CNF composite catalysts, Co_3_(PO_4_)_2_.8H_2_O, and CNF were 43.04 Ω, 84.12 Ω, and 136.19 Ω, respectively, with the Co_3_(PO_4_)_2_.8H_2_O/CNF composite having the lowest value compared to the other catalysts, indicating its fast kinetic process in HER activity. Mass loading of 0.707 mg/cm^2^ was introduced onto the prepared Co_3_(PO_4_)_2_.8H_2_O/CNF electrode to catalyze the HER under acidic conditions in a three-electrode configuration. For deep understanding of the influence of cobalt phosphate nano fibers (Co_3_(PO_4_)_2_.8H_2_O/CNF) we have also determine the mass activity (MA) of catalysts by mass loading (presented in Figure 8B) at an overvoltage of 133 mV. Co_3_(PO_4_)_2_.8H_2_O/CNF has the highest mass activity of 169.30 A g^−1^, compared to its counterparts Co_3_(PO_4_)_2_.8H_2_O (119.34 A g^−1^) and CNF (42.00 A g-^1^). Mass activity was calculated using the following equation:

$$Mass\;activity\;\left(A\;g^{-1}\right)=\frac{j\;\left(A\;{cm}^{-2}\right)}{m\;\left(g\;{cm}^{-2}\right)}.$$ (1)

The HER process proceeds through three principal steps, called the Volmer, Heyrovsky, and Tafel steps, in the acidic medium (Pentland et al., 1957; Conway and Tilak, 2002; Xie et al., 2013; Basu et al., 2017). The Volmer reaction is associated with proton absorption, which is a primary discharge step (Step 1). The Heyrovsky step is the electrochemical desorption stage (i.e., the combination of a second proton with an absorbed H atom of H_2_ gas) (Step 2). The Tafel step is a recombination step (i.e., the combination of two nearby absorbed H atoms to produce H_2_ gas) (Step 3).

$$H_{3}O^{+}+e^{-}+M\;\to \;M-H_{ads}+H_{2}O\;\left(step\;1\right)$$

$$M-H_{ads}+H_{3}O^{+}+e^{-}\to \;M+H_{2}+H_{2}O\;\left(step\;2\right)$$

$$M-H_{ads}+M-H_{ads}\;\to \;M+H_{2}\;\left(step\;3\right)$$

where H_ads_ represents a hydrogen atom chemically adsorbed on an active site of the catalyst surface (M). If the Volmer reaction is the rate-determining step, then the Tafel slope should be 120 mV dec^−1^, and for the Heyrovsky process and Tafel process, Tafel slopes of 40 and 30 mV dec^−1^ should be obtained, respectively (Xie et al., 2013; Hu et al., 2016). Therefore, combinations of steps (i.e., the Volmer–Heyrovsky or Volmer–Tafel pathways) are required to produce molecular hydrogen in a complete HER process.

**FIGURE 9:**
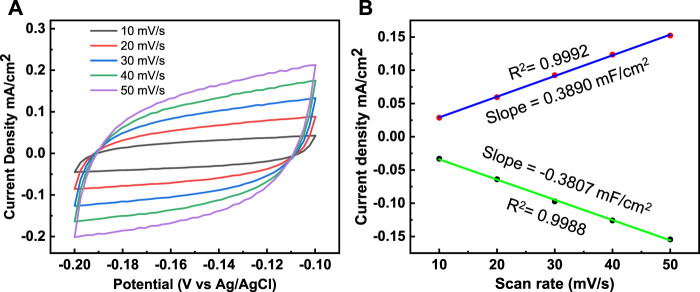
**(A)** Cyclic voltammetry curves and **(B)** the corresponding plots of J_a_ and J_c_ against the scan rate for the determination of double-layer capacitance (C_dl_) of the Co_3_(PO_4_)_2_.8H_2_O/CNF composite catalysts.

In addition, the electrochemical double layer capacitance (C_dl_) and the electrochemically active surface area (ECSA) were investigated by cyclic voltammetry (CV) performed at different sampling rates, from 10–50 mV s^−1^ (Figure 9A). C_dl_ was estimated by measuring voltammograms in a non-Faradic region, and C_dl_ was measured to determine the origin of the high HER activity of Co_3_(PO_4_)_2_.8H_2_O/CNF composite nanostructures. Both the anodic and cathodic double-layer charging currents (Ja and Jc, respectively) were calculated, and the values were plotted against the corresponding sample rates. Thus, the calculated C_dl_ for the Co_3_(PO_4_)_2_.8H_2_O/CNF composite is shown in Figure 9B and is 3.072 mF cm^−2^; the corresponding ECSA is 76.97 cm^2^, and 1.66/41.7 and 0.83/20.96 for the Co_3_(PO_4_)_2_.8H_2_O and CNF catalysts C_dl_/ECSA, respectively (Supplementary Figures S1C, D and Table 1). The Brunauer–Emmett–Teller (BET) study shows that Co_3_(PO_4_)_2_.8H_2_O/CNF has the highest surface area of 37.6 m^2^ g^−1^ compared to other constituents Co_3_(PO_4_)_2_.8H_2_O (28.2 m^2^ g^−1^) and CNF (21.4 m^2^ g^−1^); the results for these constituents are displayed in Supplementary Figure S2. The catalyst possesses excellent durability and stability after 20 h, without apparent chemical or structural deformation; XRD and FE-SEM after stability measurements are presented (Supplementary Figure S3).

## Conclusion

Co_3_(PO_4_)_2_.8H_2_O/CNF and Co_3_(PO_4_)_2_.8H_2_O were synthesized by a simple hydrothermal procedure. The structural characterizations confirmed the formation of Co_3_(PO_4_)_2_.8H_2_O/CNF with a flower-like structure attached over carbon nanofibers. The synthesized catalyst Co_3_(PO_4_)_2_.8H_2_O/CNF shows excellent performance for HER with the low overpotential (133 mV) required to generate current densities of 10 mA cm^−2^, a small Tafel slope (48 mV decade^−1^), and good stability at 24 h. The composite helps increase the high electroactive surface area, high conductivity, and vertical growth over conductive CNFs, exposing a high density of edge phosphate. This newly developed [Co_3_(PO_4_)_2_.8H_2_O/CNF] can be considered a promising electrocatalyst for HER in acidic media because of its straightforward synthetic procedure and low cost.

## Data Availability

The original contributions presented in the study are included in the article/Supplementary Material; further inquiries can be directed to the corresponding author.
