# Supplementary material for: Access to carbon nanofiber composite hydrated cobalt phosphate nanostructure as an efficient catalyst for the hydrogen evolution reaction
Source: Front Chem. 2023 Feb 23;11:1129133. doi: 10.3389/fchem.2023.1129133 (PMC9995511; doi:10.3389/fchem.2023.1129133)
Supplement: Supplementary file 1 [file DataSheet1.docx]

**Supporting Information**

**Access to carbon nanofiber composite hydrated cobalt phosphate nanostructure as an efficient catalyst for hydrogen evolution reaction**

Imtiaz Ahmed^Ϯ^, Rathindranath Biswas^Ϯ^, Rohit Sharma^Ϯ^, Vishal Burman^Ϯ^ and Krishna Kanta Haldar^Ϯ^*

^Ϯ^Department of Chemistry, Central University of Punjab, Bathinda, 151401, India.

*Author to whom correspondence should be addressed; electronic Email: [krishankant.haldar@cup.edu.in](mailto:krishankant.haldar@cup.edu.in).

**Instrumentation:**

The phase purity of the materials is identified on PANalytical Empyrean instrument through X-ray diffraction (XRD) pattern. The diffractogram is obtained in the 2θ range from 10° to 80° using Cu kα radiation source (λ = 1.5406 Ǻ) with step size of 0.004 at 45 kV. The Carl Zeiss (Merlin compact, 2014) FESEM instrument was used to perceive scanning electron microscopy (SEM) images. The compositions and oxidation states of all the samples were obtained using X-ray photoelectron spectroscopy (XPS) measurements were performed using the Thermo Scientific Inc. System equipped with a microfocus monochromatic Al Kα X-ray source of energy ~1450 e. All electrochemical measurements were performed with Metrohm Autolab (M204 multichannel potentiostat galvanostat using Nova 2.1.4 software. Brunauer-Emmett-Teller (BET) measurement was carried out with Make-Quanta Chrome instruments, Model: AutosorbiQ and ASiQwin, FTIR was performed on a Bruker Tensor 27 (Model: NEXUS-870) spectrometer in the range of 4000-600 cm^-1^ using the standard KBr disc method. The thermogravimetric analysis (TGA) results were measured with a Mettler Toledo TGA/DSC 1 thermogravimetric analyzer, under N_2_ atmosphere and a heating rate of 10 °C/min.

**Preparation of Co_3_(PO_4_)_2_.8H_2_O**

Synthesis of Co_3_(PO_4_)_2_.8H_2_O were prepared by simple co-precipitation method, 357.7 mg CoCl_2_ (1.5 mmol) was mixed in the 90 mL of deionized water followed by stirring of 5 min to form a uniform suspension of solution. Then 132.06 mg (NH_4_)_2_HPO_4_ (1 mmol) was added dropwise in 10 mL deionized water. Thereafter, 10 mL solution was tardily injected to the solution of CoCl_2_ through a micro-syringe at room temperature. Finally, the whole solution was kept stirring for 2 h at room temperature. The obtained green precipitate was collected and washed with distilled water and ethanol respectively to remove the remnant and finally the sample was dried at 70 ^o^C for further use.

**Preparation of electrodes:**

The excellent properties of Co_3_(PO_4_)_2_.8H_2_O/CNF catalyst for HER in acidic electrolyte make it promising as a catalyst for electrocatalytic water splitting. To this end, the preparation of the working electrode (WE), material ink was prepared by wisely crushing the mixture of Co_3_(PO_4_)_2_.8H_2_O/CNF using a mortar and pestle in which 5 mg of the finely ground product was taken in a 0.5 mL solution containing a mixture of ethanol (200 μL) and deionized water (300 μL) and sonicated for 30 minutes. Then, 25 μL of the binder was carefully added and sonicate again for 10 minutes thereafter 0.5 μL drop-coated over a GC (glassy carbon) electrode, and it was dried at ambient temperature for overnight. Electrochemical measurements were led by Autolab 302N modular potentiostat/galvanostat equipped with Nova 1.11 software. A single compartment cell contained a conventional three electrode assembly constituting a glassy carbon electrode (GCE, 3 mm) hosting electrocatalyst functions as the working electrode (WE), Ag/AgCl in 0.5 M H_2_SO_4_ as a reference electrode, and graphite as the counter electrode.

To gain insight into the surface accessibility and the superior HER activity of a catalyst cyclic voltammetry and electrochemically active surface area are shown in Figure 7. Electrochemically active surface areas (ECSA) were estimated as a measure of showing active site density by calculating the double-layer capacitance (*C*_dl_) from the slope determined from the linear plot anodic (*J*_a_) and cathodic (*J*_c_) current density.[5] The electrochemically active surface area (ECSA) is proportional to the electrochemical double layer capacitance (C_dl_) and the value can be calculated using the following equation:

ECSA = C_dl_/C_s_

Where C_s_ is the specific capacitance of flat working electrode and its value is 40 μF cm^-2^ per cm^2^_ECSA_ for the flat GC electrode

**Figure S1.** (a & b) Cyclic voltammetry curves and (c & d) is its corresponding plot of J_a_ and J_c_ against scan rate for the determination of double layer capacitance (C_dl_) of the Co_3_(PO_4_)_2_.8H_2_O and CNF catalysts.

**Figure S2.** BET analysis: The N_2_ adsorption-desorption isotherm of (a) CNF (b) Co_3_(PO_4_)_2_.8H_2_O and (c) Co_3_(PO_4_)_2_.8H_2_O/CNF.

**Figure S3**. (a) XRD pattern (b) FE-SEM images of Co_3_(PO_4_)_2_.8H_2_O/CNF composite catalyst after the stability measurements.

**Figure S4**. XPS survey spectra of Co_3_(PO_4_)_2_.8H_2_O/CNF composite catalyst.

**Table S1.** Comparison of OER and HER activity of Co_3_(PO_4_)_2_.8H_2_O/CNF composite catalyst with some reported Cobalt- phosphate based and CNF based catalysts in acidic medium.

| **Catalyst** | **Method of catalyst synthesis** | **Overpotential (mV)** | **Tafel slope (mV dec^-1^)** | **Electrolytes** | **References** |
| --- | --- | --- | --- | --- | --- |
| Co, Mo_2_C-CNF | Hydrothermal procedure | 128 and 206 mV | 60 mV dec^-1^ | 1M KOH | [1] |
| Mn-doped FeP/Co_3_(PO_4_)_2_ | Hydrothermal procedure | 117 mV | 44 mV dec^-1^ | 0.5M H_2_SO_4_ | [2] |
| N-doped CNF/MoS_2_ | Hydrothermal procedure | 108 mV | 61 mV dec^-1^ | N2 purged 0.5M H_2_SO_4_ | [3] |
| Co_3_(PO_4_)_2_.8H_2_O | Hydrothermal | 138 mV | 74 mV dec^-1^ | 0.5M H_2_SO_4_ | [4] |
| MoS_2_-CNF | Hydrothermal method | 207 mV | 60 mV dec^-1^ | N2 saturated 0.5M H_2_SO_4_ | [5] |
| FeCNFs-N | Electrospinning | 200 mV | 58 mV dec^-1^ | 0.5M H_2_SO_4_ | [6] |
| CoNPs-N doped CNF | Electrospinning | 159 mV | 87 mV dec^-1^ (acidic)  113 mV dec^-1^ (alkaline) | 0.5M H_2_SO_4_  1 M KOH | [7] |
| Plasma activated Co_3_(PO_4_)_2_ | Hydrothermal method | 50 mV | 35 mV dec^-1^ | 1 M KOH | [8] |
| CNF-CoP | Hydrothermal method | 83(Acidic)  191(Neutral)  138(Alkaline) | 62  mV dec^-1^ | 0.5M H2SO4  1M PBS  1 M KOH | [9] |
| **Co_3_(PO_4_)_2_.8H_2_O/CNF** | **Hydrothermal method** | **Acidic (133 mV)** | **48 mV dec^-1^** | **0.5M H2SO4** | **This Work** |

**Table S2:** PXRD Rietveld refined structural information and atomic position of Co_3_(PO_4_)_2_.8H_2_O/CNF composite catalyst.

| **Phases** | **Atoms** | **Wyckoff site** | **Positional parameters** | | | **Sym-metry** | **Occupancy** |
| --- | --- | --- | --- | --- | --- | --- | --- |
|  |  |  | **x** | **y** | **z** |  |  |
| Co_3_(PO_4_)_2_.8H_2_O | Co1  Co2  P1  O1  O2  O3  O4  O5  H1  H2  H3  H4 | 2a  4g  4i  8j  8j  8j  4i  4i  8j  8j  8j  8j | 0.00000  0.00000  0.18647  0.09779  0.10276  0.16139  0.10593  0.15820  0.04783  0.09336  0.11022  0.15786 | 0.00000  0.38840  0.50000  0.11647  0.27560  0.40063  0.50000  0.00000  0.22418  0.07098  0.28928  0.13064 | 0.00000  0.00000  0.621420  0.80306  0.27653  0.76462  0.29361  0.38220  0.28218  0.64517  0.46149  0.89297 | 2/m  2  m  1  1  1  1  1  1  1  m  m | 1.00  1.00  1.00  1.00  1.00  1.00  1.00  1.00  1.00  1.00  1.00  1.00 |
|  | Crystal system = Cubic, lattice parameters: a = 10.0210 Å, b = 13.32390 Å, c = 4.67245 Å, α = γ = 90°, β = 104.8240°, cell volume = 603.095581 Å^3^, Volumic mass density = 2.813 g/cm^3^, space group = C 1 2/m 1 (#12). | | | | | | |
| CNF (assigned to graphite) | C1  C2 | 2b  2c | 0.00000  0.33333 | 0.00000  0.066667 | 0.25000  0.25000 | ‒6m2  ‒6m2 | 1.00  1.00 |
|  | Crystal system = Hexagonal, lattice parameters: a = b = 2.4711 Å, c = 6.7582 Å, α = β = 90°, γ = 120°; cell volume = 35.7379 Å^3^, Volumic mass density = 2.232 g/cm^3^, space group = P6_3_/mmc (#194). | | | | | | |
| **Phase Fractions (%) and Bragg R-factors** | | | | | | | |
| - Cubic phase assigned to Co_3_O_4_ = 85.21 %   Bragg R-factor: 10.04 & Rf-factor: 16.1   - Monoclinic phase assigned to WO_3_ = 14.79 %   Bragg R-factor: 8.75 & Rf-factor: 19.2 | | | | | | | |
| **Rietveld R-factors** | | | | | | | |
| R_p_ = 1.43, R_wp_ = 1.81, R_exp_ = 1.73; χ^2^ = 1.10 | | | | | | | |

[1] J. Wang, R. Zhu, J. Cheng, Y. Song, M. Mao, F. Chen, Y. Cheng, Co, Mo_2_C encapsulated in N-doped carbon nanofiber as self-supported electrocatalyst for hydrogen evolution reaction, Chemical Engineering Journal 397 (2020) 125481.

[2] H. Liu, X. Peng, X. Liu, G. Qi, J. Luo, Porous Mn‐Doped FeP/Co_3_(PO_4_)_2_ Nanosheets as Efficient Electrocatalysts for Overall Water Splitting in a Wide pH Range, ChemSusChem 12 (2019) 1334-1341.

[3] F. Lai, Y.-E. Miao, Y. Huang, Y. Zhang, T. Liu, Nitrogen-doped carbon nanofiber/molybdenum disulfide nanocomposites derived from bacterial cellulose for high-efficiency electrocatalytic hydrogen evolution reaction, ACS applied materials & interfaces 8 (2016) 3558-3566.

[4] H. Singh, I. Ahmed, R. Biswas, S. Mete, K.K. Halder, B. Banerjee, K.K. Haldar, Genomic DNA-mediated formation of a porous Cu_2_(OH)PO_4_/Co_3_(PO_4_)_2_·8H_2_O rolling pin shape bifunctional electrocatalyst for water splitting reactions, RSC advances 12 (2022) 3738-3744.

[5] C. Zhang, Z. Wang, S. Bhoyate, T. Morey, B.L. Neria, V. Vasiraju, G. Gupta, S. Palchoudhury, P. Kahol, S. Mishra, MoS_2_ decorated carbon nanofibers as efficient and durable electrocatalyst for hydrogen evolution reaction, c 3 (2017) 33.

[6] I. Wang, oxide and phosphide encapsulated within N, P-doped microporous carbon nanofibers as advanced tri-functional electrocatalyst toward oxygen reduction/evolution and hydrogen evolution reactions and zinc-air batteries, J. Power Sources 367.

[7] L. Zhang, S. Zhu, S. Dong, N.J. Woo, Z. Xu, J. Huang, J.-K. Kim, M. Shao, Co nanoparticles encapsulated in porous N-doped carbon nanofibers as an efficient electrocatalyst for hydrogen evolution reaction, Journal of The Electrochemical Society 165 (2018) J3271.

[8] H. Liu, X. Liu, Z. Mao, Z. Zhao, X. Peng, J. Luo, X. Sun, Plasma-activated Co_3_(PO_4_)_2_ nanosheet arrays with Co^3+^-Rich surfaces for overall water splitting, Journal of Power Sources 400 (2018) 190-197.

[9] H. Lu, W. Fan, Y. Huang, T. Liu, Lotus root-like porous carbon nanofiber anchored with CoP nanoparticles as all-pH hydrogen evolution electrocatalysts, Nano Research 11 (2018) 1274-1284.
